# Our Dear Boy

**Published:** 1888-03

**Authors:** 


### OUR DEAR BOY.

I saw my wife pull out the bottom drawer of the old bureau, this evening, and I went softly out and wandered up and down until I knew she had shut it up and gone to her sewing. We have some things laid away in that drawer which the gold of kings could not buy, and yet they are relics which grieve us until our hearts are sore. I haven't dared look at them for a year, but I remember each article. There are two worn shoes, a little chip hat with part of the brim gone, some stockings, pantaloons, a coat, two or three spools, bits of broken crockery, a whip, and several toys. Wife, poor thing, goes to that drawer every day of her life, and prays over it, and lets her tears fall upon the precious articles, but I dare not go. Sometimes we speak of little Jack, but not often. It has been a long time, but, somehow we can't get over grieving. Sometimes, when we sit alone of an evening, I writing and she sewing, a child will call out in the street as our boy used to, and we will both start up with beating hearts and a wild hope, only to find the darkness more of a burden than ever. It is still and quiet now. I look up to the window where his blue eyes used to sparkle at my coming, but he is not there. I listen for his pattering feet, his merry shout, his ringing laugh, but there is no sound. There is no one to search my pockets and tease me for presents, and I never find the chairs turned over, the broom down, or ropes tied to the door knobs. I want some one to tease me for my knife ; to ride on my shoulder ; to lose my axe ; to follow me to the gate when I go, and be there to meet me when I come ; to call "good night" from the little bed now empty. And wife, she misses him still more. There are no little feet to wash, no prayers to say, no voice teasing for lumps of sugar, or sobbing with the pain of hurt toe ; and she would give her own life, almost, to awake at midnight and look across to the crib and see our boy there as he used to be. So we preserve our relics, and when we are dead we hope strangers will handle them tenderly even if they shed no tears over them —*Cape Ann Advertiser.*

